# Elevation in Sphingomyelin Synthase Activity Is Associated with Increases in Amyloid-Beta Peptide Generation

**DOI:** 10.1371/journal.pone.0074016

**Published:** 2013-08-20

**Authors:** Jen-Hsiang T. Hsiao, YuHong Fu, Andrew F. Hill, Glenda M. Halliday, Woojin Scott Kim

**Affiliations:** 1 Neuroscience Research Australia, Sydney, New South Wales, Australia; 2 Department of Biochemistry and Molecular Biology, Bio21 Institute, University of Melbourne, Parkville, Victoria, Australia; 3 Mental Health Research Institute of Victoria, Parkville, Victoria, Australia; 4 School of Medical Sciences, University of New South Wales, Sydney, New South Wales, Australia; Nathan Kline Institute and New York University School of Medicine, United States of America

## Abstract

A pathological hallmark of Alzheimer’s disease (AD) is the presence of amyloid-beta peptide (Aβ) plaques in the brain. Aβ is derived from a sequential proteolysis of the transmenbrane amyloid precursor protein (APP), a process which is dependent on the distribution of lipids present in the plasma membrane. Sphingomyelin is a major membrane lipid, however its role in APP processing is unclear. Here, we assessed the expression of sphingomyelin synthase (SGMS1; the gene responsible for sphingomyelin synthesis) in human brain and found that it was significantly elevated in the hippocampus of AD brains, but not in the cerebellum. Secondly, we assessed the impact of altering SGMS activity on Aβ generation. Inhibition of SGMS activity significantly reduced the level of Aβ in a dose- and time dependent manner. The decrease in Aβ level occurred without changes in APP expression or cell viability. These results when put together indicate that SGMS activity impacts on APP processing to produce Aβ and it could be a contributing factor in Aβ pathology associated with AD.

## Introduction

Alzheimer’s disease (AD) is a neurodegenerative disorder characterized clinically by dementia and pathologically by the presence of amyloid-beta peptide (Aβ) plaques in brain regions associated with memory and learning. Aβ is derived from a sequential cleavage of the amyloid precursor protein (APP) by β- and γ-secretases through the β-secretase pathway [1]. The monomeric form of Aβ, predominantly composed of 40 or 42 amino acids, has the propensity to form oligomeric and fibril complexes that impact on multiple processes that eventually lead to AD neurodegeneration [2–5]. An alternative processing of APP is through the α-secretase pathway, in which α-secretase along with γ-secretase cleave APP to form the non-amyloidogenic peptide P3. While the regulation of Aβ generation is complex, increasing evidence indicate that the distribution of lipids in the plasma membrane affects how APP is processed and subsequently the level of Aβ produced [6].

A lipid anomaly in AD brain, described as “lipid granule accumulation”, was first observed by Alois Alzheimer as one of three neuropathological features of AD brain, along with senile plaques and neurofibrillary tangles [7]. In recent years, genome wide association studies have revealed a number of genes involved in lipid membrane dynamics to be strongly linked to typical late onset AD (LOAD) [7,8]. The strongest risk factor for LOAD identified thus far is the E4 isoform of apolipoprotein-E (apoE4), which plays a crucial role in lipid transport in the central nervous system [9]. Despite intense research into the biological function of apoE, the precise mechanism by which apoE4 increases AD risk remains to be fully elucidated, although there is evidence showing subtle yet significant differences in the lipid binding property of apoE4 when compared to other isoforms [10–12]. ABCA7, which belongs to a lipid transporting family, is another candidate gene for LOAD [13]. ABCA7 regulates both lipid efflux and Aβ accumulation, indicating that the two processes are intrinsically linked [14,15].

There are regions in the plasma membrane that are enriched in lipids and these so-called lipid rafts are characterized by high concentrations of cholesterol, sphingolipids and saturated phospholipids. Other regions of the plasma membrane (non-lipid rafts) are composed of mainly unsaturated phospholipids and low concentrations of cholesterol and sphingolipids. APP localized in lipid rafts is preferentially cleaved by β-secretase to form Aβ; whereas APP localized in non-lipid rafts are predominantly cleaved by α-secretase to form non-amyloidogenic products [16–20]. Sphingomyelin is a major sphingolipid present in the plasma membrane and is highly enriched in lipid rafts. The exact role of sphingomyelin in the plasma membrane remains unclear although it is thought to play a role in maintaining membrane structure and possibly in signal transduction [21]. When accumulated in high concentrations sphingomyelin causes Niemann–Pick disease (NPD) and irreversible neurological damage [22]. Increases in sphingomyelin level in NPD are strongly correlated with increases in Aβ production, which is thought to contribute to NPD neurodegeneration [23].

The exact role of sphingomyelin in APP processing associated with AD is unclear. In the current study we assess whether the level of SGMS, the gene responsible for sphingomyelin synthesis, is altered in AD brain, and we investigate the impact of altering SGMS activity on APP processing and Aβ generation using CHO cells that stably express human APP695.

**Figure 1:**
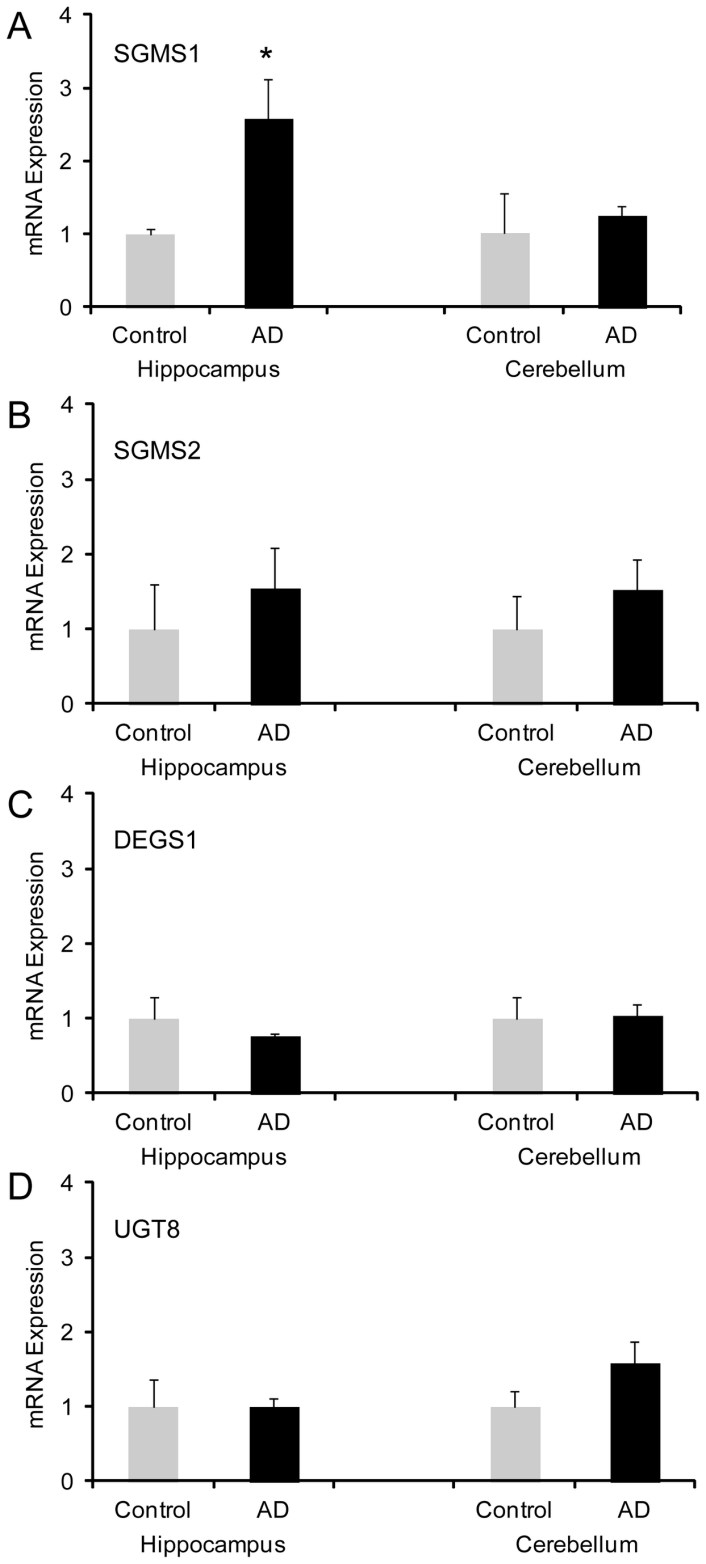
Expression of sphingolipid synthesis genes in AD brain. RNA was isolated from the hippocampus and cerebellum of control and AD brain tissues and the expression of sphingomyelin synthase 1 (SGMS1) (A), sphingomyelin synthase 2 (SGMS2) (B), lipid desaturase (DEGS1) and UDP glycosyltransferase 8 (UGT8) was measured by qPCR. Data represent mean (n=6) + SE, ∗*p* < 0.05.

## Materials and Methods

### Brain tissues

Human brain tissues were obtained from the Sydney Brain Bank at Neuroscience Research Australia (http://neura.edu.au/sydneybrainbank) and the NSW Tissue Resource Centre at the University of Sydney (http://sydney.edu.au/medicine/pathology/trc/) under institutional ethics approvals. Whole brains were sliced into blocks and freshly frozen and stored at -80°C. Tissue samples were obtained from six cases of clinically and pathologically-verified AD and six age- and gender-matched controls without significant neuropathology, as previously described [10,24]. The postmortem delay for all twelve cases was 27 h or less. Details for each case are provided in Table 1. Approximately 50 mg of brain tissue from anatomically specified regions was collected using a 3-mm stainless steel biopsy needle while the brain slices were still frozen (on a bed of dry-ice).

**Table 1:** Demographics of control and Alzheimer’s disease cases.

**Case ID**	**Gender**	**Age**	**PMI (h)**	**Brain pH**	**Clinical cause of death**
Control 1	Male	82	23	6.4	Multiple organ failure
Control 2	Male	69	13	6.74	Myocardial infarction
Control 3	Male	79	8	6.65	Probable pulmonary embolism
Control 4	Female	93	21	6.96	Cardiac failure
Control 5	Female	85	23	6.44	Pneumonia
Control 6	Female	78	11	6.3	Respiratory failure
AD 1	Male	83	27	6.26	Cardiovascular accident
AD 2	Male	73	20	6.51	Bronchopneumonia
AD 3	Male	69	15	6.73	Colon cancer
AD 4	Female	84	9	6.32	Aspiration pneumonia
AD 5	Female	83	7	6.20	Circulatory collapse
AD 6	Female	83	23	5.88	Uraemia

### Cell Culture and treatment

Chinese hamster ovary (CHO) cells which stably express the human APP695 (CHO-APP) [25] were cultured in 12-well plates in RPMI 1640 media containing 10% fetal calf serum, 2 mM glutamine, 100 IU/ml penicillin, 100 μg/ml streptomycin and 7.5 µg/ml puromycin at 37°C in humidified air containing 5% CO_2_. Cell culture media and additives were obtained from Invitrogen (Melbourne, Australia) unless stated otherwise. CHO-APP cells were treated with tricyclodecan-9-xanthogenate (D609) or N-butyl-deoxynojirimycin (NB-DNJ) (Sigma-Aldrich, Sydney, Australia) and cells and media were collected for gene expression and Aβ analyses. siRNA transient transfection was performed using Lipofectamine RNAiMAX and Opti-MEM I (Invitrogen) following the manufacturer’s protocol. Briefly, CHO-APP cells were seeded at 40% confluence in 12-well plates using antibiotic-free media. SGMS1 siRNA and scramble siRNA (control) were designed using the BLOCK-iT RNAi designer program (www.invitrogen.com/rnai). siRNAs were added to the cells (n=3 wells each) at 50 pmol/well for 48 h, and mRNA and media were collected for gene expression and Aβ analyses. To assess transfection efficiency a second 12-well plate was prepared and cells were transfected with fluorescently labelled BLOCK-iT reagent (Invitrogen), supplied with the kit, using the same transfection procedure. This confirmed >95% transfection efficiency as assessed by fluorescence microscopy. The effectiveness of knockdown was measured by qPCR, as described below, using SGMS1 primers f: GTCGACATTCCCAACCCTGA and r: GAAGAGTCTTGCCCCACTCC, and the housekeeper gene GAPDH primers, f: GGAGAAGGCTGGGGCCCACT and r: GGTGGTGCAGGACGCATTGCT.

**Figure 2:**
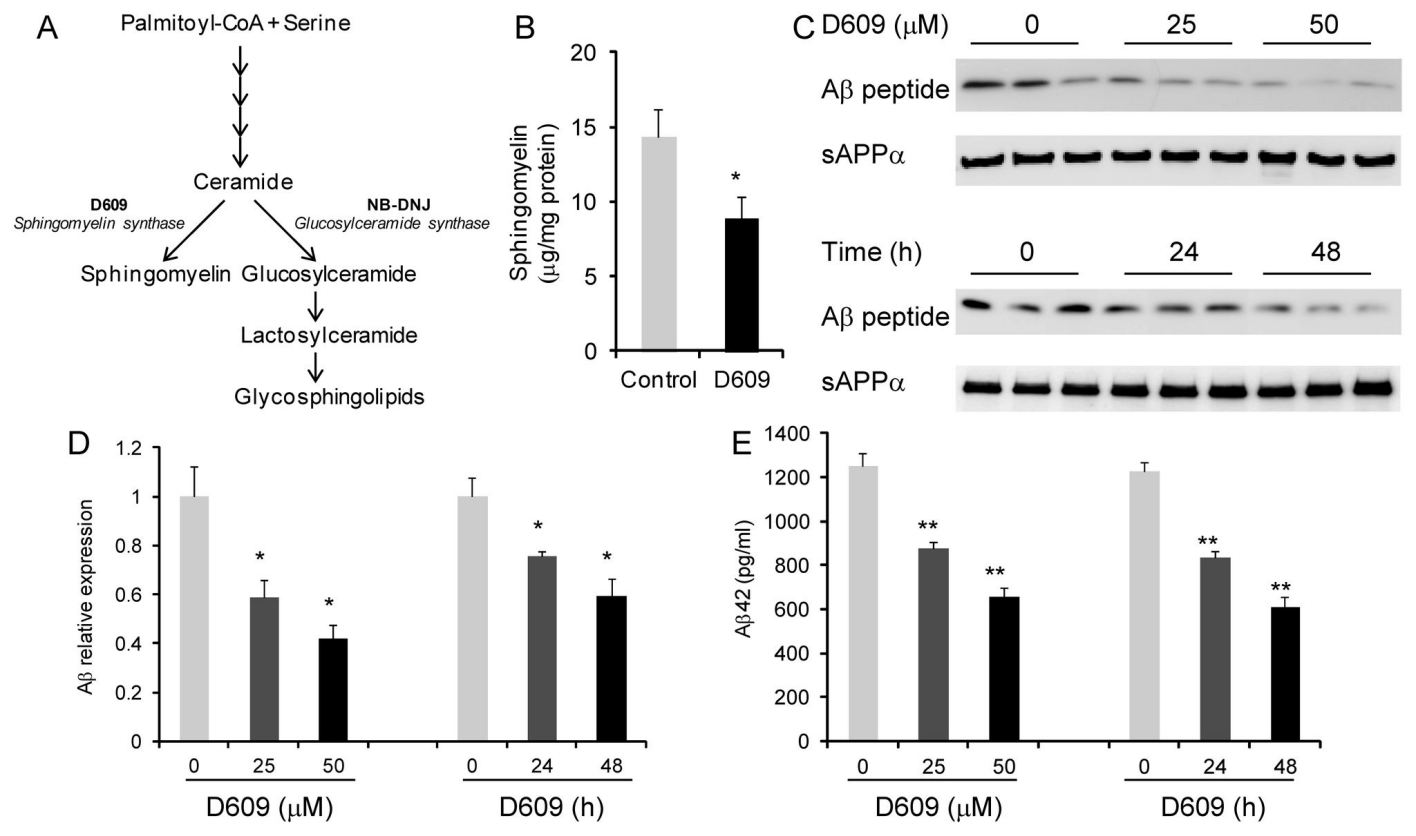
Impact of altering SGMS activity on Aβ generation. (A) Sphingomyelin and glycosphingolipid synthesis pathway. (B) CHO-APP cells were treated with the SGMS inhibitor D609 (50 μM) and sphingomyelin measured by an enzymatic assay. (C) CHO-APP cells were treated with increasing concentration of D609 for 48 h or 50 μM D609 for 0, 24 and 48 h and secreted Aβ and sAPPα were measured by western blotting. (D) Relative optical density of Aβ bands was quantified using ImageJ software. (E) Aβ42 was also measured by ELISA. Data represent mean (n=3) + SE, ∗*p* < 0.05, ∗∗*p* < 0.005.

### RNA isolation, reverse transcription and quantitative real-time PCR

RNA was isolated using TRIzol reagent (Invitrogen) following the manufacturer’s protocol. All procedures were carried out using RNase-free reagents and consumables. Five micrograms of RNA was reverse transcribed into cDNA using Moloney-murine leukemia virus reverse transcriptase and random primers (Promega, Madison, Wisconsin, USA) in 20 μl reaction volume. Quantitative real-time PCR assays were carried out using a Mastercycler ep realplex S (Eppendorf, North Ryde, NSW, Australia) and the fluorescent dye SYBR Green (Bio-Rad), following the manufacturer’s protocol. Briefly, each reaction (20 μl) contained 1x mastermix, 1x SYBR Green, 5 pmol of primers, and 1 μl of cDNA template. Amplification was carried out with 40 cycles of 94°C for 15 s and 60°C for 1 min. Gene expression was normalized to the housekeeper gene β-actin. A no-template control was also included. The level of expression for each gene was calculated using the comparative threshold cycle (Ct) value method and the formula 2^-ΔΔCt^ (where ΔΔCt = ΔCt sample - ΔCt reference).

**Figure 3:**
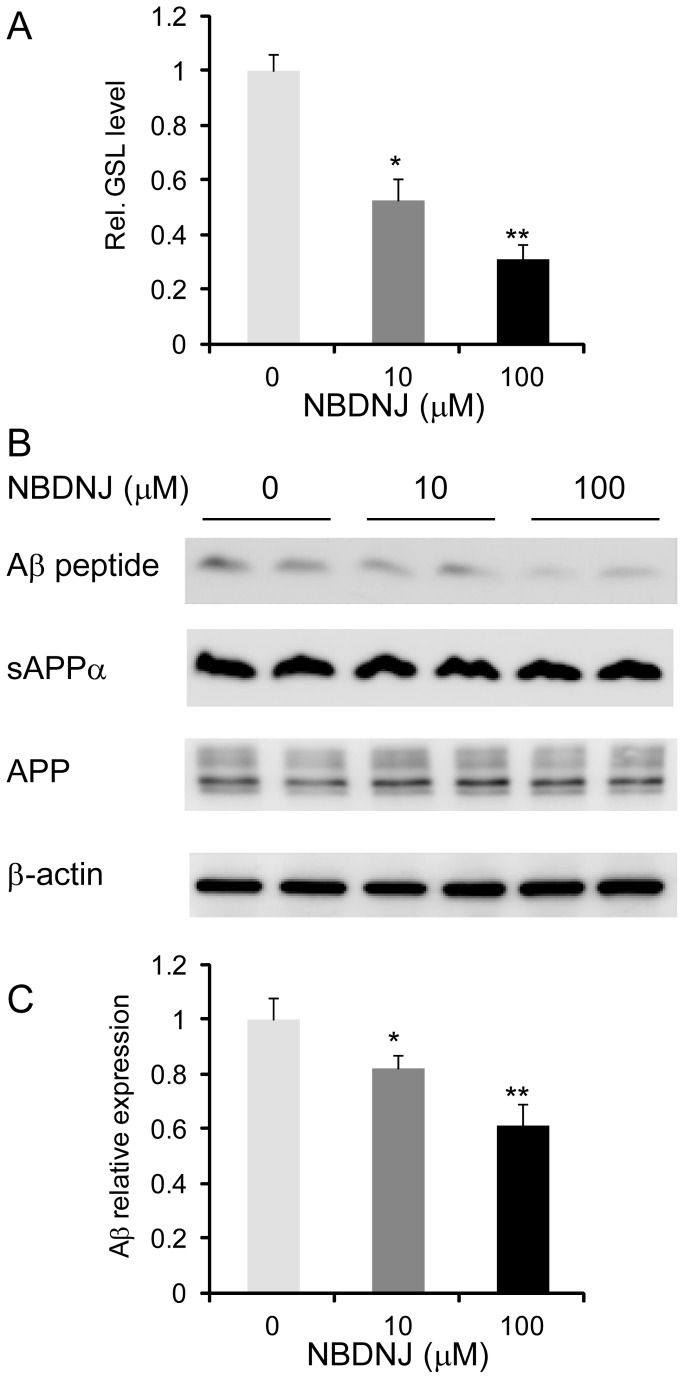
Effect of altering GSL level on Aβ generation. (A) CHO-APP cells were treated with the GSL synthesis inhibitor NB-DNJ and GSL measured by HPLC. (B) CHO-APP cells were treated with NB-DNJ and secreted Aβ and sAPPα and cellular APP measured by western blotting. The housekeeper protein β-actin shows equal protein loading. (C) Relative optical density of Aβ bands was quantified using ImageJ software. Data represent mean (n=3) + SE, ∗*p* < 0.05, ∗∗*p* < 0.005.

**Figure 4:**
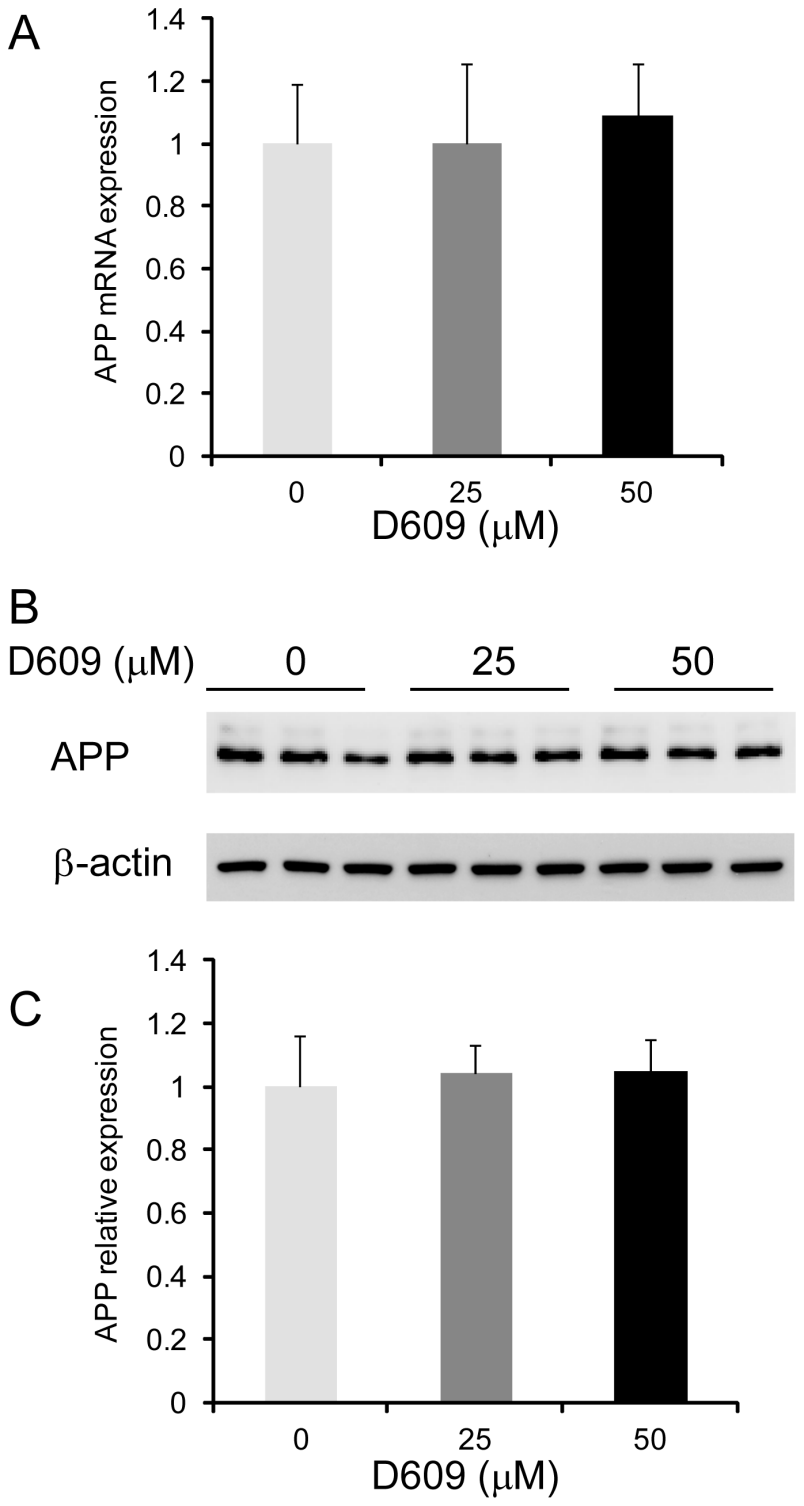
Effect of D609 on APP expression. CHO-APP cells were treated with increasing concentration of D609 for 48 h and the expression of APP was measured at mRNA level by qPCR (A) and protein level by western blotting (B). The housekeeper protein β-actin shows equal protein loading. (C) Relative optical density of APP protein bands was quantified using ImageJ software. Data represent mean (n=3) + SE.

### Lipid analysis

Analysis of cell lysates for sphingomyelin was based on a published method [26]. Sphingomyelin measurement involved a four-step enzymatic process, whereby sphingomyelinase (SMase) (Cat. No. S-7651; Sigma) hydrolyzed SM to phosphorylcholine and N-acylsphingosine. Subsequently, alkaline phosphatase (Cat. No. P5521; Sigma) generated choline from the phosphorylcholine, which in turn was used to generate hydrogen peroxide in a reaction catalyzed by choline oxidase (Cat. No. C5896; Sigma). The final step detected liberated hydrogen peroxide using Nethyl-N-(2-hydroxy-3-sulfopropyl)-3,5-dimethoxyaniline, sodium salt (DAOS) (Cat. No. OC06; Dojindo Molecular Technologies, Japan), 4-aminoantipyrine (4-AAP) (Cat. No. A4382; Sigma), and horseradish peroxidase (HRP) (Cat. No. P1139; Sigma) to generate a blue dye. The reaction buffer components were prepared in 0.05 M Tris-HCl, 0.66 mM calcium chloride, pH 8. Enzyme concentrations in a 10 ml reaction buffer were as follows: 5 units of SMase, 100 units of alkaline phosphatase, 5 units of choline oxidase, and 200 units of HRP. 4-aminoantipyrine and DAOS concentrations were both 0.73 mM. Five microliters of cell lysate was added to 100 μl of reaction buffer, and after 45 min of incubation at 37^°^C, the absorption was measured at 595 nm using a spectrophotometric plate reader (BioRad, Model 550). A standard curve was prepared using aliquots of a stock sphingomyelin solution (1 mg/ml) prepared by dissolving 10 mg of sphingomyelin in 10 ml of 2% (w/v) Triton X-100 in ethanol.

Cellular glycosphingolipids (GSL) were analyzed as described previously using normal phase HPLC [18,27]. Briefly, cells were washed in PBS and extracted in chloroform/methanol (2: 1 volume). Ceramide glycanase was used to hydrolyze total neutral and charged GSL and the resultant GSL-derived glycans fluorescently labeled with 2-aminobenzamide and analyzed as a single sample. Total glycan peak area was calculated and values for treated cells were presented relative to untreated cells.

### Western Blotting

CHO-APP cells cultured in 12-well plates were rinsed with cold PBS and lysed in lysis buffer (20 mM Tris-HCl, pH 7.5, 150 mM NaCl, 1 mM EDTA, 1% Nonidet P-40, 0.5% deoxycholate, 0.1% SDS, and protease and phosphatase inhibitors). Bicinchoninic acid protein assays were performed on lysates and equal amounts of protein were separated on SDS-PAGE gels and transferred onto 0.45 µm nitrocellulose membranes at 100 volts for 30 min. Membranes were blocked overnight at 4°C in PBS containing 5% nonfat dry milk and probed with primary antibodies. The membranes were washed three times in PBS containing 0.1% Tween 20 and then incubated with horseradish peroxidase-conjugated secondary antibodies. Signals were detected using enhanced chemiluminescence and x-ray films (GE Healthcare). The signal intensity was quantified using NIH ImageJ software. Western blotting of secreted Aβ was carried out as previously described [25]. Briefly, Aβ in the culture medium was separated on 12% SDS-PAGE gels and transferred onto 0.2 µm nitrocellulose membranes at 65 volts for 17 min. Membranes were boiled in PBS for 5 min, probed with WO2 monoclonal antibody followed by rabbit anti-mouse horseradish peroxidase-conjugated secondary antibody and ECL detection applied as described above.

### ELISA

The concentration of Aβ42 in cell culture supernatants was determined using ELISA kits (BioSource International, San Francisco, CA, USA) following the manufacturer’s instructions. Culture supernatants were diluted 1:10 in 55 mM NaHCO_3_, pH 9.0, and all standards and samples were assayed in triplicate.

### Cell viability assay

CHO-APP cells were seeded in 96-well plates at 50% confluence and were treated with increasing concentration of D609 for 24 h. Cell viability assay was carried out using a MTT assay kit (Promega) following the manufacturer’s protocol. Briefly, culture media was removed, 15 μl of dye solution containing MTT added and incubated at 37°C for 2 h. The media was then discarded, 100 μl of solubilization solution added and incubated at 37°C for 1 h. The absorbance of the cell lysates was measured at 570 nm. Cell morphology was also assessed using a light microscope and images were captured under phase contrast (20×).

### Statistical analysis

The human tissue samples were examined in hexaplicate (n=6). CHO-APP experiments were performed in triplicate (n=3) or hexaplicate (n=6) as specified in figure legends. Data presented are expressed as mean + SE shown by the error bars. Statistical significance was analyzed using the two-tailed Student’s *t* test with a *p* < 0.05 considered significant.

## Results

### Expression of SGMS in AD brain

To gather clues as to what role sphingomyelin may play in amyloid-β generation associated with AD, we measured the expression of SGMS1 and SGMS2 (two isoforms of sphingomyelin synthase), in AD brain tissue. We prepared tissue samples from six clinically and pathologically confirmed cases of AD and six age- and gender-matched AD free controls, as previously described [24,28]. We analyzed two regions of the brain, the hippocampus (severely affected by AD) and cerebellum (largely unaffected by AD). The expression of SGMS1, but not SGMS2, was significantly increased in the hippocampus of AD brain (Figure 1A,B). Neither were significantly altered in the cerebellum (Figure 1A,B). We also measured the expression of two other sphingolipid synthesis genes, i.e. lipid desaturase (DEGS1, converts dihydroceramide to ceramide) and UDP glycosyltransferase 8 (UGT8, converts ceramide to galactosylceramide) and found that neither were significantly altered in AD brain (Figure 1C,D). These results indicate that sphingomyelin could be associated with AD pathology.

**Figure 5:**
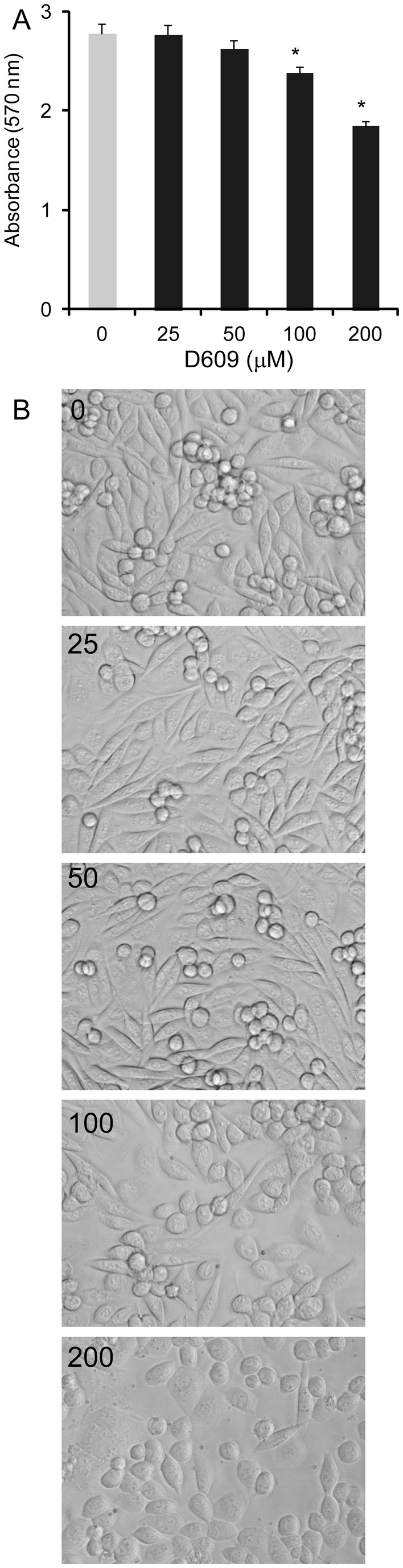
Cell viability with D609 treatment. CHO-APP cells were treated with increasing concentration of D609 for 48 h and the cell viability assessed by MTT assay (A) and morphology under a phase-contrast microscope (20×) (B). Data represent mean (n=6) + SE.

### Impact of altering SGMS activity on amyloid-β generation

Since SGMS1expression is altered in AD hippocampus, we then investigated whether SGMS1 activity affects APP processing and Aβ generation in CHO cells that stably express the human 695-amino acid APP (CHO-APP). To alter the SGMS activity we treated the cells with the SGMS inhibitor D609 (Figure 2A) and confirmed a decrease in the cellular sphingomyelin level (Figure 2B). We then measured Aβ secreted from the cells by western blotting and ELISA. Inhibition of SGMS activity significantly reduced the level of Aβ in a dose- and time-dependent manner (Figure 2C). Secreted sAPPα was also measured and it was unaltered with D609 (Figure 2C). The signal intensity of the monomeric Aβ detected by western blotting indicated that D609 inhibited Aβ production by 25–58% (Figure 2D). The ELISA measurements showed that the level of Aβ42 in the D609-treated cells was reduced by 30–50% (Figure 2E), which is consistent with the western blotting results. To further verify the link between SGMS1 activity and Aβ levels we used siRNA technology to knockdown SGMS1 expression and measure Aβ levels. CHO-APP cells were transfected with either SGMS1 siRNA (targeted to SGMS1 transcript) or scramble siRNA (control). After 48 h of incubation the cells were harvested and mRNA extracted and the expression of SGMS1 was measured by qPCR. The siRNA knockdown significantly reduced SGMS1 mRNA levels by 46%±4.1% (mean±SE) as determined using triplicate samples from two independent experiments (*P*<0.05). We also collected the media from the same samples and measured the extracellular Aβ levels by ELISA and showed that the Aβ levels were significantly reduced by 31%±4.5% (*P*<0.05). These results clearly indicate that SGMS1 activity impacts on Aβ production. Since altering sphingomyelin level may inadvertently alter GSL level (an alternative pathway for ceramide, see Figure 2A), we also assessed the impact of altering GSL level on Aβ generation by treating CHO-APP cells with NB-DNJ. NB-DNJ inhibits ceramide glucosyltransferase, the first step in GSL synthesis pathway (Figure 2A). We showed that NB-DNJ significantly reduced GSL level (Figure 3A) and suppressed Aβ production in a dose-dependent manner (Figure 3B,C). We also showed that secreted sAPPα and cellular APP levels were not significantly altered with GSL synthesis inhibition (Figure 3B).

### SGMS activity regulates APP processing and not production

To determine if the decrease in Aβ generation observed was due to changes in APP processing or simply a reduction in APP production, we treated CHO-APP cells with D609 at concentrations of 25 and 50 μM and measured the expression of APP at both mRNA and protein levels by qPCR and western blotting. We found that neither mRNA (Figure 4A) nor protein levels of APP (Figure 4B,C) were altered with D609, indicating that the decrease in Aβ generation was caused by changes in APP processing and not by a reduction in APP production.

### Cell viability assay of D609

To determine if D609 had any cytotoxic effect on CHO-APP cells that could have affected the Aβ level, we treated CHO-APP cells with increasing concentration of D609 and the cell viability and morphology were assessed. At the concentrations used in the Aβ generation studies, i.e. 25 and 50 μM, the cell viability and morphology were not significantly affected (Figure 5A,B). However, at higher concentrations of D609, i.e. 100 and 200 μM, the cell viability was significantly reduced and the cell morphology was altered, i.e. cells became rounded (Figure 5A,B).

## Discussion

This report represents a study into the impact of SGMS activity on APP processing and Aβ generation associated with AD. We have shown that the expression of the gene responsible for sphingomyelin synthesis, SGMS1, is significantly elevated in the hippocampus of AD brains, one of the brain regions targeted early by AD. The expression of SGMS1 in the cerebellum, a region of the brain that is largely unaffected by the disease, was unaltered in AD brains. These data reinforce recent findings showing that the level of sphingomyelin, as measured by high performance liquid chromatography-mass spectrometry, is significantly elevated in the entorhinal cortex, but not in the cerebellum, of AD brains [29]. The entorhinal cortex and the adjacent hippocampus form a close-knit circuit that is crucial for autobiographical and episodic memory formation and consolidation, and both regions are affected early in AD. Furthermore, a recent study in the APP/PS1 mouse model of AD showed that sphingomyelin present in the frontal cortex lipid raft increased with age correlating strongly with the severity of Aβ pathology [30]. Since SGMS1 expression and sphingomyelin levels are elevated in AD affected brain regions, sphingomyelin levels may contribute to the disease process. We therefore tested whether altering the SGMS activity could affect APP processing to produce Aβ. When the SGMS activity was inhibited the production of Aβ decreased significantly in a dose- and time-dependent manner; the Aβ level decreased as much as ~58%. We showed that the decrease in Aβ generation observed was clearly due to changes in APP processing and not due to simply reduction in the overall APP production nor loss in cell viability. Interestingly, only SGMS1 expression, not SGMS2, was altered in both AD hippocampus and J20 mouse hippocampus. SGMS1 is responsible for converting the bulk of ceramide to sphingomyelin in the lumen of the trans Golgi [31] and therefore plays a key role in the maintenance of cellular level of sphingomyelin. It is unclear whether SGMS2 participates in the de novo synthesis of sphingomyelin [32].

We and others have previously shown that glycosphingolipids present in the plasma membrane regulate APP processing to generate Aβ. Overwhelming data show that when cellular glycosphingolipid level was reduced by means of various inhibitors, Aβ generation was decreased. The inhibition of Aβ generation was demonstrated in multiple cell systems including isolated primary human neurons, human neuroblastoma SH-SY5Y cells and CHO cells expressing human APP [20,33]. Conversely, when ganglioside, a glycosphingolipid, was exogenously added to cultured cells the level of Aβ increased [20]. It is therefore clear that cellular sphingolipid levels impact on APP processing and Aβ generation.

Sphingolipids, including sphingomyelin and cerebroside, are highly enriched in membrane lipid rafts and changes in their levels may affect the activities of β- and γ-secretases residing there. In vitro studies have shown that cerebrosides stimulate β-secretase (BACE) activity to increase Aβ generation [34]. It is not known whether sphingomyelin exerts the same effect on β-secretase. However, sphingomyelin has been shown to increase the activity of purified γ-secretase and the proteolysis of C100Flag, an APP fusion protein [35], possibly providing an explanation for the decrease in Aβ level we observed in CHO-APP cells treated with D609. In a different study, sphingomyelin level was shown to be regulated by Aβ via activation of sphingomyelinase, the enzyme that degrades sphingomyelin, the process of which was dependent on γ-secretase activity [4]. Another possible explanation for the link between sphingomyelin and amyloidogenesis comes from the fact that sphingomyelin traffics between the plasma membrane and endosomes [36] and endosomal accumulation of sphingomyelin impairs degradation of APP-CTF, which enhances amyloidogenesis [37]. While the exact causation has not been proved, data from our study and others strongly indicate that SGMS activity regulates APP processing to produce Aβ. Furthermore, SGMS activity may provide a new target for developing therapeutic avenues for controlling Aβ generation and consequently AD neurodegeneration.
